# Improving the effectiveness of psychological interventions for depression and anxiety in the cardiac rehabilitation pathway using group-based metacognitive therapy (PATHWAY Group MCT): study protocol for a randomised controlled trial

**DOI:** 10.1186/s13063-018-2593-8

**Published:** 2018-04-03

**Authors:** Adrian Wells, Kirsten McNicol, David Reeves, Peter Salmon, Linda Davies, Anthony Heagerty, Patrick Doherty, Rebecca McPhillips, Rebecca Anderson, Cintia Faija, Lora Capobianco, Helen Morley, Hannah Gaffney, Gemma Shields, Peter Fisher

**Affiliations:** 1 School of Psychological Sciences, Faculty of Biology, Medicine and Health, Rawnsley Building, Manchester Royal Infirmary, The University of Manchester, Oxford Road, Manchester, M13 9WL UK; 2 Greater Manchester Mental Health NHS Foundation Trust, Rawnsley Building, Manchester Royal Infirmary, Oxford Road, Manchester, M13 9WL UK; 3 NIHR School for Primary Care Research, Manchester Academic Health Science Centre, The University of Manchester, Williamson Building, Oxford Road, Manchester, M13 9PL UK; 4 Institute of Psychology, Health and Society, University of Liverpool, Waterhouse Building, Block B, Brownlow Street, Liverpool, L69 3GL UK; 5 The Royal Liverpool and Broadgreen University Hospitals NHS Trust, Prescot Street, Liverpool, L7 8XP UK; 6 Centre for Health Economics, Division of Population Health, Health Services Research and Primary Care, School of Health Sciences, Faculty of Biology, Medicine and Health, The University of Manchester, Jean McFarlane Building, Oxford Road, Manchester, M13 9PL UK; 7 School of Medical Sciences, Core Technology Facility, The University of Manchester, Grafton Street, Manchester, M13 9NT UK; 8 Central Manchester Foundation Trust, Manchester Royal Infirmary, Oxford Road, Manchester, M13 9WL UK; 9 Department of Health Sciences, University of York, Seebohm Rowntree Building, York, YO10 5DD UK; 10 School of Biological Sciences, Division of Neuroscience and Experimental Psychology, The University of Manchester, Oxford Road, Manchester, M13 9PL UK; 11 School of Health Sciences, Division of Psychology and Mental Health, The University of Manchester, Oxford Road, Manchester, M13 9PL UK

**Keywords:** Metacognitive therapy, rumination, worry, anxiety, depression, cardiac rehabilitation, group therapy, psychological intervention, heart disease

## Abstract

**Background:**

Anxiety and depression are prevalent among cardiac rehabilitation patients but pharmacological and psychological treatments have limited effectiveness in this group. Furthermore, psychological interventions have not been systematically integrated into cardiac rehabilitation services despite being a strategic priority for the UK National Health Service. A promising new treatment, metacognitive therapy, may be well-suited to the needs of cardiac rehabilitation patients and has the potential to improve outcomes. It is based on the metacognitive model, which proposes that a thinking style dominated by rumination, worry and threat monitoring maintains emotional distress. Metacognitive therapy is highly effective at reducing this thinking style and alleviating anxiety and depression in mental health settings. This trial aims to evaluate the effectiveness and cost-effectiveness of group-based metacognitive therapy for cardiac rehabilitation patients with elevated anxiety and/or depressive symptoms.

**Methods/Design:**

The PATHWAY Group-MCT trial is a multicentre, two-arm, single-blind, randomised controlled trial comparing the clinical- and cost-effectiveness of group-based metacognitive therapy plus usual cardiac rehabilitation to usual cardiac rehabilitation alone. Cardiac rehabilitation patients (target sample *n* = 332) with elevated anxiety and/or depressive symptoms will be recruited across five UK National Health Service Trusts. Participants randomised to the intervention arm will receive six weekly sessions of group-based metacognitive therapy delivered by either cardiac rehabilitation professionals or research nurses. The intervention and control groups will both be offered the usual cardiac rehabilitation programme within their Trust. The primary outcome is severity of anxiety and depressive symptoms at 4-month follow-up measured by the Hospital Anxiety and Depression Scale total score. Secondary outcomes are severity of anxiety/depression at 12-month follow-up, health-related quality of life, severity of post-traumatic stress symptoms and strength of metacognitive beliefs at 4- and 12-month follow-up. Qualitative interviews will help to develop an account of barriers and enablers to the effectiveness of the intervention.

**Discussion:**

This trial will evaluate the effectiveness and cost-effectiveness of group-based metacognitive therapy in alleviating anxiety and depression in cardiac rehabilitation patients. The therapy, if effective, offers the potential to improve psychological wellbeing and quality of life in this large group of patients.

**Trial registration:**

UK Clinical Trials Gateway, ISRCTN74643496, Registered on 8 April 2015.

**Electronic supplementary material:**

The online version of this article (10.1186/s13063-018-2593-8) contains supplementary material, which is available to authorized users.

## Background

Cardiac rehabilitation (CR) services aim to improve heart disease patients’ health and quality of life, and reduce the risk of recurrent cardiac events [1]. Approximately 96,000 patients attend CR annually in the UK across 324 CR programmes at an annual total cost of approximately £42 million [1–3]. Emotional distress is highly prevalent among CR patients. National CR data demonstrate that 37% of patients starting CR have clinically significant levels of anxiety and/or depressive symptoms and 40% of these patients have mixed anxiety and depression, which further increases the burden of distress [1]. Post-traumatic stress disorder is also common with 12% of acute coronary syndrome patients developing this disorder following hospitalisation with a myocardial infarction or unstable angina [4].

Distressed patients are at greater risk of death and further cardiac events [5, 6], have a poorer quality of life [7], and use more healthcare resources [8–10], leading to greater National Health Service (NHS) costs [11]. Distress can also delay or prevent patients returning to work [12]. Therefore, emotional distress is a significant problem for CR patients, the NHS and society.

In 2010, the UK Department of Health implemented the CR commissioning pack to improve CR services. The pack details a comprehensive service specification with a seven-stage care pathway from patient presentation, referral and assessment through to long-term maintenance [13]. Although psychological assessment and support are advocated throughout this pathway and in other key NHS policies [14–16], CR patients with anxiety and depression are not being effectively treated; indeed, only 19% of group CR programmes include a psychological component, 2% of patients receive individual psychological interventions, and no manualised psychological interventions for depression and/or anxiety are available for general use [1]. Following CR, 24% of patients still have elevated anxiety and 13% have depressive symptoms, with rates rising to 26% and 16%, respectively, 9 months later [1]. Pharmacological and psychological treatments that have been evaluated in these patients have limited effects on psychological outcomes [17–21] and health-related quality of life (HRQoL) [22], and no improvement in cardiovascular outcomes [17, 21]. Given the limitations of existing treatments and CR services, it is imperative that effective psychological interventions for depression and anxiety are developed and integrated into the CR pathway to improve clinical outcomes, patient quality of life and cost-effectiveness.

An evidence-based model from mental health – the metacognitive model [23, 24] – provides the basis for a potentially effective and practicable treatment for depression and anxiety in CR patients. In this model, a maladaptive style of thinking and coping maintains symptoms across a wide range of emotional problems, including depression and anxiety. The model comprises three processes, namely (1) repetitive, difficult-to-control thinking (worry and/or rumination), (2) focusing attention on potential threats (e.g. thoughts, physical sensations, emotions), and (3) maladaptive attempts to control unwanted thoughts (e.g. avoidance, reassurance-seeking, alcohol/substance misuse). These metacognitive processes are maintained by two types of underlying metacognitive beliefs, specifically (1) positive beliefs about the usefulness of worry, rumination, threat monitoring and other coping strategies (e.g. “Worrying about my health will help prevent future illness”, “Constantly focusing on my body helps to keep me safe”) and (2) negative beliefs about the uncontrollability of thoughts and their damaging effects on the body and mind (e.g. “I cannot control my rumination”, “Worry will damage my health”). The metacognitive model is transdiagnostic, wherein a small number of processes and beliefs maintain all forms of emotional distress. Consistent with this model, the same processes and beliefs are associated with depression and anxiety in patients with a diverse range of physical health conditions, including heart disease [25–27].

Metacognitive therapy (MCT) modifies the processes and beliefs that maintain distress using a range of well-specified strategies and techniques (see [28] for treatment manual). In mental health settings, MCT for depression and anxiety disorders has been evaluated in case series, as well as in uncontrolled and controlled trials [29–35]. A recent meta-analysis found MCT to be highly effective in treating depression and anxiety disorders. Large within-group effect sizes were found across all trials from pre- to post-treatment, with treatment gains maintained at follow-up. In controlled trials, treatment gains following MCT were large when compared to wait-list controls and cognitive behaviour therapy [36].

There are three main reasons why MCT should generalise to heart disease patients. First, as the metacognitive model is transdiagnostic, disparate symptoms of distress are addressed with the same core treatment strategies; this makes it particularly well-suited to heart disease patients who often have mixed anxiety and depression. Second, the same strategies are used to treat distress irrespective of medical diagnosis, which makes it compatible with a CR pathway design, where a range of heart disease patients undertake the same CR programmes and where 80% of patients have at least one other long-term condition [1]. Finally, MCT focuses on modifying cognitive *processes* (e.g. reducing worry duration) rather than on cognitive *content* (e.g. reality testing worries), which is the focus of cognitive behaviour therapy. Therefore, it is amenable to working with the realistic negative thoughts that often occur in the context of heart disease, e.g. thoughts about the possibility of recurrent cardiac events and the functional limitations associated with poor physical health.

### Aims

The primary aim of the PATHWAY Group-MCT trial is to evaluate the effectiveness of group-based MCT (Group-MCT) plus usual CR compared to usual CR alone in alleviating depression and/or anxiety in patients attending CR. Secondary aims are (1) to evaluate the impact of Group-MCT on secondary outcomes including post-traumatic stress, metacognitive beliefs, and HRQoL, (2) to evaluate the cost-effectiveness of Group-MCT, and (3) to obtain qualitative data to develop an account of the barriers and enablers to the trial and to the intervention, and to interpret evidence of effectiveness, including processes that might underpin or compromise effectiveness or explain its heterogeneity.

## Methods/Design

### Design

**Fig. 1:**
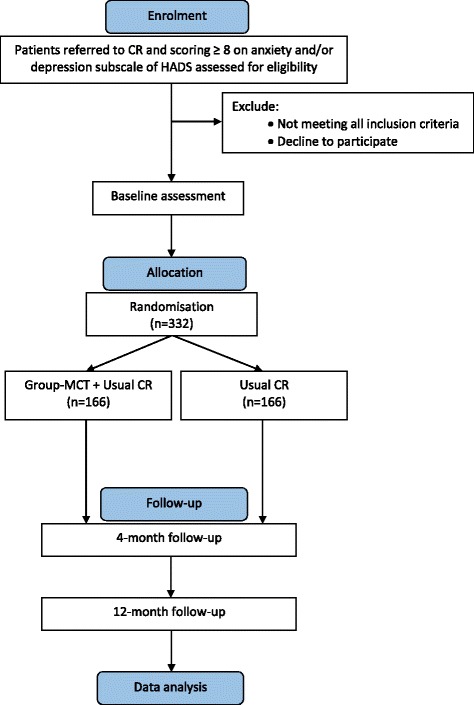
Trial flow diagram. *CR* cardiac rehabilitation, *HADS* Hospital Anxiety and Depression Scale, *MCT* metacognitive therapy

**Fig. 2:**
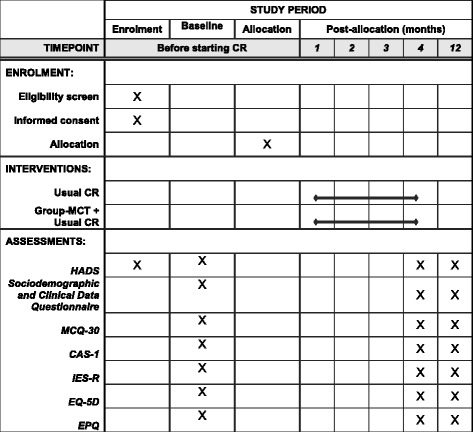
Schedule of enrolment, interventions and assessments. *CR* cardiac rehabilitation, *Group-MCT* group-based metacognitive therapy, *HADS* Hospital Anxiety and Depression Scale, *MCQ-30* Metacognitions Questionnaire 30, *CAS-1* Cognitive Attentional Syndrome Scale, *IES-R* Impact of Event Scale-Revised, *EPQ* Economic Patient Questionnaire

The PATHWAY Group-MCT trial is a multicentre, two-arm, single-blind randomised controlled trial with 4- and 12-month follow-up comparing Group-MCT plus usual CR with usual CR alone. Treatment as usual (CR alone) is the chosen comparator because there is currently no benchmark treatment for distress in this group, existing treatments have limited effects and this is the first test of group MCT in this patient population. There are qualitative and economic evaluations embedded within the trial. An overview of the study process is provided in Fig. 1. A schedule of enrolment, interventions and assessments is provided in Fig. 2 and a populated Standard Protocol Items: Recommendations for Intervention Trials (SPIRIT) Checklist is provided in Additional file 1.

### Trial population

The trial population are heart disease patients referred to CR services at five NHS Trusts (University Hospital of South Manchester NHS Foundation Trust, Central Manchester University Hospitals NHS Foundation Trust, East Cheshire NHS Trust, Stockport NHS Foundation Trust, and Pennine Acute Hospitals NHS Trust).

### Inclusion and exclusion criteria

Inclusion criteria:

1. Meets Department of Health and/or British Association for Cardiac Prevention and Rehabilitation CR eligibility criteria (acute coronary syndrome, revascularisation, stable heart failure, stable angina, implantation of cardioverter defibrillators/cardiac resynchronisation devices, heart valve repair/replacement, heart transplantation and ventricular assist devices, adult congenital heart disease, other atypical heart presentation).
2. A score of ≥ 8 on either the depression or anxiety subscale of the Hospital Anxiety and Depression Scale (HADS) [37]
3. Aged 18 years or older
4. A competent level of English language skills (able to read, understand and complete questionnaires in English)

Exclusion criteria:

1. Cognitive impairment which precludes informed consent or ability to participate
2. Life expectancy of less than 12 months
3. Acute suicidality
4. Active psychotic disorders
5. Current drug or alcohol abuse
6. Antidepressant or anxiolytic medications initiated in the previous 8 weeks
7. Concurrent psychological intervention for emotional distress

### Recruitment and randomisation

Patients referred to the CR programme at each site are routinely sent a National Audit of Cardiac Rehabilitation assessment pack [1], which includes the HADS [37]. Patients return the pack to the CR team either at an initial assessment appointment or by post. CR staff will screen the medical notes of patients scoring eight or above on either the anxiety or depression subscale of the HADS to determine if they meet the trial inclusion and exclusion criteria. Patients deemed eligible for the trial will be provided with information about the study and a member of the CR team will seek an expression of interest from the patients.

Eligible and interested patients will be contacted by a research assistant to arrange a suitable time and location to meet prior to the patients starting the CR programme (e.g. patients’ homes or NHS Trusts). Research assistants will take written consent and administer baseline questionnaires. After baseline assessments, patients will be randomised via telephone link to the Manchester Academic Health Science Centre Clinical Trials Co-ordination Unit (MAHSC-CTU). Patients will be allocated to trial arms in a 1:1 ratio via a minimisation algorithm (incorporating a random component) to maximise balance between the two arms on sex, HADS anxiety and depression scores [38], and hospital site. A member of the research team will inform the participants of their allocation. Research assistants who are collecting assessment data will be blind to treatment allocation, as will the chief investigator (AW) and trial statistician (DR).

### Trial conditions

#### Usual CR

Participants in the control group will be offered the usual CR programme at their site. The content and delivery of usual CR varies slightly across the participating sites but all offer group-based programmes delivered in either hospital or community settings. Group-based CR runs weekly over 8–10 weeks and comprises a number of components, including exercise, lifestyle and medical risk factor management, and health behaviour change. There is some limited psychosocial input (e.g. one-off talks on stress management and relaxation techniques), although one site offers a 4-week stress management course as part of CR. Four of the sites also offer a home-based programme to a small number of patients. Home-based CR is tailored to each patient’s needs but comprises similar components to group-based CR, including an exercise programme that can be undertaken at home or in the community as well as educational components.

#### Group-MCT plus usual CR

The intervention group will receive group-based MCT in addition to the usual CR at their site. The group intervention will consist of six weekly sessions delivered by two trained CR professionals (physiotherapists, nurses, occupational therapists) or research nurses (depending on site) over 1–1.5 h. The therapists will be guided by a treatment manual to maximise treatment adherence. The aim is to deliver the intervention to groups of between three and ten participants, but this will vary depending on recruitment and attrition rates. The aims of the intervention are to help participants develop knowledge that can facilitate control of worry, rumination and attention, and to modify the metacognitive beliefs that maintain these unhelpful patterns of thinking [33]. There are eight major treatment techniques that will be used across the six sessions, namely (1) formulation, (2) socialisation, (3) the Spatial Attentional Control Exercise, (4) detached mindfulness, (5) worry and rumination postponement, (6) modifying metacognitive beliefs about the uncontrollability and danger of worry and rumination, (7) a ‘helpful behaviours prescription’, and (8) individual treatment summaries. Sessions include group discussions, experiential learning and homework tasks that participants will be expected to complete between sessions. Participants will complete a set of ‘belief thermometers’ at the start of each session, which measure three core metacognitive beliefs. The thermometers will be used as a clinical tool for participants and therapists to monitor change over the course of the intervention. Treatment will be discontinued at any time for a participant requesting it.

#### Therapist training and supervision

Therapists will initially receive 2 days of workshop training delivered by the developer of MCT (AW), which will include didactic teaching, role plays, discussion and studying of the treatment manual. This will be followed by supervised practise in delivering the intervention to a pilot group of volunteers and a further 1-day workshop, which will address difficulties the therapists experienced when delivering the intervention. Therapists’ delivery of the intervention will be monitored by listening to audio-recordings of Group-MCT sessions and they will receive supervision as necessary throughout the trial.

#### Therapist competence and treatment adherence

Therapist competence will be evaluated by audio-recording a random sample of 10% of treatment sessions (where consent is provided by all participants in a group). The sessions will be rated by independent MCT experts using a competency checklist. Inter-rater Kappa coefficients for competency ratings will be reported. Adherence to the manual will be assessed using an adherence checklist completed by the therapists at each session.

### Data collection

Participants will complete assessments at three time-points – baseline (pre-CR), 4 months post-randomisation (4-month follow-up) and 12 months post-randomisation (12-month follow-up). Research assistants will administer baseline assessments (see ‘Recruitment and randomisation’ above). Participants will have a number of options for completing the 4- and 12-month assessments either by post, or administered by research assistants over the telephone or face-to-face at participants’ homes or NHS trust. Participants will receive shopping vouchers in return for completing the assessments.

#### Measures

The following self-report outcome measures will be completed at each assessment point:

##### Primary outcome measure

- The HADS [37] comprises 14 items measuring symptoms of anxiety (7 items) and depression (7 items). Respondents rate their emotional state over the previous week on a four-point (0–3) scale. Possible scores for each subscale range from 0 to 21 and can be categorised as normal (0–7), mild (8–10), moderate (11–14) or severe (15–21). The subscales of the English version of the HADS have good internal consistency (Cronbach’s alphas ranging from 0.72 to 0.93) [39].

##### Secondary outcome measures

- The Impact of Event Scale-Revised (IES-R) [40] is a 22-item measure of trauma-related symptoms associated with specific life events. The instructions have been tailored for this trial so participants rate how distressing each ‘difficulty’ has been over the past week with respect to their ‘heart event which occurred recently’. Respondents rate each item on a five-point scale ranging from 0 (‘not at all’) to 4 (‘extremely’). The IES-R yields a total score ranging from 0 to 88. The IES-R subscales have good internal consistency (Cronbach’s alphas ranging from 0.79 to 0.91) as well as good test–retest reliability ranging from 0.51 to 0.94) [40].
- The Metacognitions Questionnaire 30 (MCQ-30) [41] measures domains of metacognition assessed by 30 items across five subscales, namely (1) positive beliefs about worry (e.g. “Worrying helps me cope”); (2) negative beliefs about thoughts concerning uncontrollability and danger (e.g. “When I start worrying I cannot stop”); (3) low cognitive confidence (e.g. “My memory can mislead me at times”); (4) beliefs about the need to control thoughts (e.g. “Not being able to control my thoughts is a sign of weakness”), and (5) cognitive self-consciousness (e.g. “I pay close attention to the way my mind works”). Respondents rate how much they “generally agree” with the statements presented on a four-point scale (1 = do not agree; 2 = agree slightly; 3 = agree moderately; 4 = agree very much). The MCQ-30 also yields a total score ranging from 30 to 120. The MCQ-30 total score and the ‘negative beliefs about thoughts concerning uncontrollability and danger’ subscale will be used in the trial, while recognising the overlap between these. The total score has been selected as the MCQ-30 has been shown to have a bifactor structure, consisting of a dominant general factor alongside the five sub-scales [42]. The uncontrollability and danger subscale has been selected as it is the primary mechanism targeted in MCT. These two scales will also be used in mediator and moderator analyses. The MCQ-30 possesses good internal consistency (Cronbach’s alphas 0.72 to 0.93 for individual subscales) [41] as well as good convergent validity and acceptable test–retest reliability [38, 41, 43].
- The EQ-5D-5L [44] is a widely-used, standardised, generic measure of HRQoL that assesses an individual’s health across five dimensions, namely mobility, self-care, usual activities, pain/discomfort, and anxiety/depression. Each dimension has five response categories ranging in severity from “no problems” to “extreme problems”. The EQ-5D-5L is a valid and reliable measure in the cardiovascular population [45].

##### Process measure

- The Cognitive Attentional Syndrome-1r (CAS-1r) has been adapted from the original 16-item version of the CAS-1 [28] for this study. The CAS-1r assesses individuals’ metacognitive strategies and knowledge. The first six items evaluate the extent to which an individual engages in worry, rumination and other strategies (e.g. “How much time in the last week have you found yourself dwelling on or worrying about your problems?”). The final four items assess an individual’s metacognitive beliefs. The CAS-1r is scored on a scale from 0 (none of the time) to 100 (all of the time), with higher scores indicating more use of metacognitive strategies or greater conviction in metacognitive beliefs. The original CAS-1 has adequate internal consistency (Cronbach’s alpha = 0.86) [42]. The CAS-1r will be used for mediator and moderator analyses.

##### Sociodemographic and clinical data questionnaire

- A questionnaire will be used to collect sociodemographic information (age, sex, ethnic origin, marital status, living arrangements, employment status, educational attainment) and clinical data (height, weight, smoking status, alcohol use, age at first cardiovascular event, history of cardiovascular events, comorbidities, past and current medications for anxiety or depression, past or current psychological therapies).

##### Health economic measures

- The primary measure of health benefit is quality-adjusted life years (QALYs), which will be estimated from the EQ-5D-5L (see above [44]) and published utility tariffs [46–48]. The EQ-5D-5L is the measure recommended by the UK National Institute of Health and Care Excellence for use in economic evaluations of new interventions [49]. At the end of the trial, a data request will be submitted to NHS Digital to obtain data on hospital-based service use and the use of mental health services covering all assessment points (baseline, 4-month and 12-month follow-up). To supplement these data, an Economic Patient Questionnaire will assess participants’ use of inpatient and outpatient services and data on non-hospital-based health and social care use at all assessment points.

#### Qualitative methods

The perspectives of both the therapists delivering Group-MCT and participants in the trial will be evaluated. All therapists will be interviewed longitudinally. Interviews conducted before training in Group-MCT will explore their understanding of the psychological needs of CR patients and of whether and how CR addresses these needs. During training, therapists will be prompted about their understanding of Group-MCT, their experiences of training and their expectations of delivering Group-MCT. After therapists have completed training and delivered Group-MCT, they will be prompted about their experiences of delivering it and about whether and how their knowledge and understanding of Group-MCT has altered.

Participants assigned to the intervention group will be sampled purposively to include ranges of age, psychological distress and diagnoses; sampling will stop when theoretical saturation is reached [50, 51]. Based on previous research, it is anticipated that approximately 30 participants in the intervention group will be interviewed. Interviews before Group-MCT will explore participants’ emotional needs and experiences since their index event, any interaction of emotional needs with clinical care, and their reactions and expectations upon being offered Group-MCT. Participants who complete Group-MCT will then be interviewed about their experience of the intervention, including its content and delivery, and any relevance of the intervention to their emotional distress. Any participants who do not complete Group-MCT will be asked for interviews to explore their experience of the intervention and their reasons for not completing it.

As a check on whether, and how, being offered the intervention has influenced participants’ accounts, participants assigned to the control group will also be interviewed, purposively sampled to include ranges of age, psychological distress and diagnoses; it is anticipated that approximately 10 participants will be interviewed. They will be interviewed only once, will have varying exposures to CR, and will be prompted to talk about their emotional experiences since the index event and any interaction of emotional needs with clinical care. Patients who declined to participate but who consent to qualitative interviews will also be invited for an interview, and will explore their reasons for declining.

Interviews with therapists and participants will be semi-structured and conversational in style, using an interview guide. They will be on NHS premises or in the case of participants in their homes, as participants wish. Interviews will be anonymously transcribed verbatim for analysis.

### Outcomes

The primary outcome of the trial is severity of anxiety and depressive symptoms at 4-month follow-up as measured by the HADS total score. Secondary outcomes include severity of anxiety and depressive symptoms at 12-month follow-up, and HRQoL, severity of post-traumatic stress symptoms and conviction in metacognitive beliefs at both 4- and 12-month follow-up.

### Sample size calculation

The trial is designed with 90% power to detect a standardised mean difference (SMD) between trial arms of 0.4 in HADS total score at 4-month follow-up. The target SMD of 0.4 is in the middle of the range of effect sizes reported for other forms of psychological interventions for depression [17] and is conservative.

An internal pilot trial with 52 participants was conducted first to ascertain the feasibility of recruitment to the trial and to collect data on which to calculate a definitive sample size calculation for the main trial. The internal pilot was assessed at 4-month follow-up. Relative to the standard deviation in HADS total score at baseline, the target SMD of 0.4 represents a difference between trial arms of 2.2 points on the HADS total score. Based on an observed 35% attrition rate at 4 months (participants not returning questionnaires), a correlation between HADS total score at baseline and follow-up of 0.5, an average group size of three and assumed intracluster correlation coefficient of 0.05, a total of 332 participants will need to be recruited, including the 52 already recruited to the pilot trial as no substantial changes were made to the procedures or trial instruments following the pilot.

## Analyses

The trial will be analysed using quantitative, qualitative and economic methods.

### Quantitative analysis

All analyses will follow intention-to-treat principles and a pre-specified plan. Analysis will be undertaken using Stata version 14 [52] and an alpha level of 5%. Analysis of covariance within a regression framework will examine differences in outcomes between trial arms. The primary analysis will use the baseline values of each outcome, the minimisation variables (hospital site, sex, HADS anxiety and depression scores), and other pre-specified variables predictive of outcomes as covariates. The analysis will account for the clustering of patients within CR or CR plus Group-MCT groups. Appropriate sensitivity analyses will be conducted to assess the robustness of the results to missing data (e.g. using multiple imputation methods), non-normal outcome distributions and choice of covariates.

If a significant intervention effect on the primary outcome (HADS total score at 4 months) is detected, a structural equation modelling framework will be used to explore whether this effect was mediated by changes in metacognitions (the basic assumption of the Group-MCT intervention).

### Qualitative analysis

The data from participants and therapists will be analysed separately. Each analysis will draw upon a pluralist qualitative approach, initially informed by constant comparison and grounded theory principles, to explore what is present and what is noticeably absent in the data [51]. We will go beyond line-by-line coding of content, to attend to how participants talked, and to consider data in the context of the whole interview, successive interviews for each participant, the participants’ clinical and institutional context, and the emerging analysis. Knowledge of each participant’s primary outcome (HADS) will be part of the context for interpreting their accounts. Data will be considered descriptively at first, with a more interpretative approach developing as analysis proceeds. Procedurally, analysis will draw on constant comparison, as we iterate between the developing analysis and new interviews and revisit earlier interviews in light of the developing analysis.

Analysis will be developed and tested by discussion amongst a core analytic team consisting of a sociologist and clinical psychologists with experience of qualitative methods and expertise in MCT, who will read all transcripts, and by periodic discussion in the broader study team who will read selected transcripts. Differences of interpretation during discussions will alert the team to potential competing explanations. Deviant cases will be highlighted to test and develop the analysis. As well as consensus validity [53], the emerging analysis will be judged according to its ‘catalytic validity’ [54], whereby it should have potential real-life implications, and theoretical validity, whereby it should have implications for existing theory [54]. Metacognitive theory [23, 24] will not be imposed as a structure for analysis, but findings will be related to it.

### Economic analysis

The primary cost effectiveness analysis will use an intention-to-treat approach to estimate total costs and QALYs for the 4-month follow-up period of the trial from the perspective of the NHS and Social Care. The key outcome of the economic evaluation will be the incremental cost effectiveness ratio, a joint measure of cost and health benefit. Each item of resource use will be multiplied by the relevant published national unit cost for that item. National unit costs are published annually by the Department of Health [55] and the Personal Social Services Research Unit, University of Kent [56]; the price year will be the most recent year for which national unit costs are available (expected to be 2017/2018). QALYs gained from baseline to follow-up will be estimated from the EQ-5D-5L and published utility tariffs [46–48].

All missing cost and utility data will be treated as missing at random and multiple imputations will be used to impute values for missing data for each follow-up period. The imputation procedure will use predictive mean matching and sequential chained equations. The variables included in the imputation models will be selected on the basis of descriptive and regression analyses of the pooled baseline and follow-up data to identify potential predictors of the utility, follow-up and cost measures [57]. Cost data will be imputed by category rather than as a total so that all available data are used to inform the imputed values.

Regression analysis will be used to estimate the net costs and net QALYs of Group-MCT plus usual CR compared to usual CR alone. The regression models will include covariates that may affect the costs or QALYs. The covariates will be derived from discussion with the trial team and analysis of the trial data, which will identify variables for inclusion in a model via a stepwise approach. The net cost and QALY estimates will be bootstrapped to generate 10,000 pairs of net costs and QALYs. These will then be used to estimate the probability that the trial intervention is cost-effective. Cost-effectiveness planes and cost-effectiveness acceptability curves will be plotted. Net benefit statistics will be estimated; these approaches require that net QALYs are revalued using a monetary value that reflects decision-makers willingness to pay to gain one QALY. The range of £0 to £30 k will be used in line with current estimates of the willingness-to-pay threshold implicit in National Institute of Health and Care Excellence decisions [58–63].

Sensitivity analyses will explore uncertainty, including the impact of using alternative sources for costs and utility tariffs, approaches to missing data, key outcomes and time horizons.

The economic evaluation will be reported in line with the Consolidated Health Economic Evaluation Reporting Standards statement [64].

## Trial management and oversight arrangements

This trial forms part of a larger research programme funded by the National Institute of Health Research under its Programme Grants for Applied Research scheme (RP-PG-1211 20,011). The programme is overseen by an independent programme steering committee, which meets every 6 months to provide expert advice, supervise the overall programme on behalf of the National Institute of Health Research and the sponsor, and monitor progress against agreed milestones. A programme executive committee, comprising the chief investigator, co-investigators, the core project team and other relevant parties, meets quarterly. Notwithstanding the legal obligations of the sponsor and chief investigator, the executive committee has operational responsibility for the conduct of the trial, including monitoring overall progress to ensure adherence to the protocol and for taking appropriate action to safeguard participants and the quality of the trial. A programme management group comprising the chief investigator and core project team meet weekly to oversee the day-to-day management of the programme. There is also a service user advisory group, which meets at least every 6 months and provides advice and feedback on a range of trial-related activities, e.g. reviewing study documents.

MAHSC-CTU is a clinical trials unit with UK Clinical Research Collaboration registration, which provides full data management and trial monitoring services for the trial as well as having an advisory role with regards to trial conduct and adherence to regulatory requirements.

## Safety reporting

Health professionals delivering the Group-MCT intervention will monitor participants attending the group sessions for any potential adverse events or serious adverse events. Any events deemed to be related to the intervention will be reported to the research team and reviewed by a designated sub-investigator who is not blind to treatment allocation. Any serious adverse events will be reported to the ethics committee, the programme steering committee and the sponsor’s Research and Innovation Manager within 7 days of the event. Adverse events and serious adverse events will be reviewed on a quarterly basis at the programme’s executive committee meetings.

## Dissemination

The trial results will be published in peer-reviewed journals and these will be made freely available and online wherever possible. The findings will also be presented at national and international clinical-academic cardiovascular, health economic and psychological therapies conferences as well as general public health conferences, regional conferences and forums, and public involvement events. Important protocol modifications will be communicated to the research ethics committee, trial registry, clinical trials unit, steering committee and all relevant parties.

## Discussion

Anxiety and depression are common among patients with heart disease, with 37% reporting significant anxiety and/or depressive symptoms [1]. Currently available drug and psychological treatments have only small effects on distress and quality of life, and no benefits to physical health in this patient population [17–22]. Furthermore, the needs of heart disease patients are not being met currently within UK NHS CR services. Given the limitations of existing CR services and treatment options, there is an urgent need for new, effective psychological interventions for depression and anxiety to be integrated into the CR pathway in order to improve clinical outcomes. MCT [28] has been empirically tested in mental health settings through case series and uncontrolled and controlled trials [29–36], where it has consistently demonstrated large post-treatment reductions in depression and anxiety and high recovery rates. The PATHWAY Group-MCT trial will establish the effectiveness of Group-MCT in alleviating anxiety and depression in CR patients. The study also will provide quantitative data for modelling the psychological mechanisms of therapeutic change and qualitative data to understand barriers and enablers to the trial and to the intervention, and participants’ and therapists’ experiences of MCT. Finally, to aid decision-making, data on healthcare service use and health status will be used to assess whether Group-MCT is a potentially cost-effective intervention.

## Trial status

The PATHWAY Group-MCT trial is currently recruiting participants and recruitment is predicted to continue until February 2018.

## Additional file

Additional file 1: Standard Protocol Items: Recommendations for Intervention Trials (SPIRIT) Checklist. (DOC 121 kb)

## Abbreviations

CAS-1r: Cognitive Attentional Syndrome-1r
CR: cardiac rehabilitation
Group-MCT: group-based metacognitive therapy
HADS: Hospital Anxiety and Depression Scale
HRQoL: health-related quality of life
IES-R: Impact of Event Scale-Revised
MAHSC-CTU: Manchester Academic Health Science Centre Clinical Trials Co-ordination Unit
MCQ-30: Metacognitions Questionnaire 30
MCT: metacognitive therapy
NHS: National Health Service
QALY: quality-adjusted life year
SMD: standardised mean difference
